# An Equivalence Testing Approach to the Calculation of Time to Stabilization Following Drop Landing on Force Plates in Athletic Populations

**DOI:** 10.3390/s26154702

**Published:** 2026-07-24

**Authors:** Andrew Kates, Ming-Chang Tsai, Marc Klimstra

**Affiliations:** 1 Faculty of Health, University of Victoria, Victoria, BC V8W 2Y2, Canada; klimstra@uvic.ca; 2 Canadian Sport Institute Pacific, Whistler, BC V9E 2C5, Canada

**Keywords:** force plates, ground reaction forces, time to stabilization, dynamic postural stability, drop landing, equivalence testing, TOST

## Abstract

**Highlights:**

This study proposes a novel equivalence testing approach (TOST) for calculating time to stabilization (TTS) from force plate-derived ground reaction forces following drop landing, offering a statistically principled alternative to fixed-threshold methods with improved reliability and compatibility with short trial lengths.

**What are the main findings?**

- TOST with equivalence scaling factors ≥ 11 yielded behaviourally stable TTS estimates with consistently ‘good’ reliability (ICC(2,5) ≈ 0.76–0.88) and low measurement error (SEM ≈ 0.035–0.038 s) in the vertical axis.
- TOST showed high agreement with the unprocessed (RAW) method while demonstrating superior within-session reliability and substantially lower measurement error than previously reported for existing methods.

**What are the implications of the main findings?**

- TOST provides a statistically principled framework for future investigation of dynamic postural stability, enabling shorter and more practical testing protocols for large groups of athletes.
- The use of the sequential average (SA) method as a measure of dynamic postural stability should be questioned given the strong evidence of trial length dependence; it is recommended that researchers consider TOST as a replacement for RAW in the vertical axis.

**Abstract:**

Current time to stabilization (TTS) calculation methods use a signal-to-threshold approach, whereby post-landing ground reaction forces (GRFs) are compared against fixed thresholds derived from a stable period. This approach does not formally test statistical equivalence and may be sensitive to arbitrary processing and threshold selection. This study proposes a new equivalence-based method, utilizing two one-sided tests of equivalence (TOST) to calculate TTS following a drop landing in athletic populations. Twenty-six elite freestyle ski athletes (males = 14, females = 12; age = 22.36 ± 2.14 years) performed five trials of a 50 cm drop landing on force plates. TOST was defined as the first time point at which a 50 ms rolling window, advanced one sample at a time, achieved and maintained statistical equivalence to a pre-defined equivalence region. The TOST method was tuned by systematically assessing the effect of the equivalence width (k) and persistence (dwell) on pre-defined criteria emphasizing differences in TTS due to parameter changes, reliability (intraclass correlation coefficient; ICC(2,5)), and measurement error (SEM). The final parameters were selected as the lowest k and highest dwell values resulting in ‘good’ reliability (ICC ≥ 0.75) within the behaviourally stable region, or the highest observed ICC where this threshold was not met. TOST was then compared to previous TTS calculation methods (no-processing (RAW), sequential average (SA) and third-order polynomial (TOP)) using Bland–Altman and by comparing the ICC and SEM. For the vertical GRF, equivalence scaling factors (k) ≥ 11 yielded behaviourally stable TTS estimates with consistently ‘good’ reliability (ICC(2,5) = 0.78 [95% CI: 0.61,0.89]) and low SEM (0.035–0.038 s), regardless of the dwell value. Anterior-posterior and medio-lateral GRFs showed low marginal changes in response to parameters at k ≥ 10 and 13, respectively, but the reliability was poor to moderate and, in the ML axis, fell below zero under some parameter settings, reflecting the limited horizontal demand of the task. Recommendations for TOST are therefore made for the vertical axis only. Comparisons between methods revealed high agreement between TOST and RAW in the vertical axis, while TOST demonstrated higher reliability. SA produced TTS values on a markedly different scale consistent with trial length dependence, while TOP systematically overestimated TTS with significant proportional bias, indicating that neither could be used interchangeably with TOST. This framework provides a statistically principled alternative to fixed threshold-based TTS definitions and, unlike trial length-dependent methods such as SA and TOP, requires only a trial long enough to capture a stable reference period, permitting shorter trials better suited to frequent testing with large groups of athletes.

## 1. Introduction

Time to stabilization (TTS) is a measure of dynamic postural stability (DPS) following landing from a drop or a jump, defined as the time required for ground reaction forces (GRFs) to return to a baseline or stable state [1]. A recent scoping review exploring TTS measurement in athlete populations included 74 studies reporting a total of 128 TTS outcome measures from 111 landing tasks, clearly highlighting sport scientists’ interest in utilizing this metric to understand neuromuscular function [2]. TTS has shown the ability to differentiate between individuals with existing lower limb impairments at both the knee [3] and ankle [4] and can be improved via neuromuscular training programs in both injured [5] and healthy [6,7] athletes. More broadly, landing strategy and lower limb kinematics are closely linked to injury risk during landing tasks [8], underscoring the value of objective measures of landing performance.

Despite strong empirical and theoretical support for TTS as a measure of DPS in athletes, there is great variation in TTS values between studies and no gold standard or consistent basis for comparison [9]. The current most common calculation methods can be broadly categorized and identified by their signal processing approach: unprocessed (RAW) or modelled by a sequential average (SA) or a third-order polynomial (TOP). In each case, the processed signal is considered stable once it either meets or remains within a pre-defined threshold. Within each of these methods, differences in input signal (V = vertical, AP = antero-posterior, ML = medio-lateral), signal processing, threshold calculation, definition of stability, and trial length are known to affect the TTS outcome [9]. Between-study differences in any of these parameters may explain the variation in TTS values and contribute to conflicting results such as a lack of expected group differences [10] or variable/unexpected responses to neuromuscular training programs [11].

Beyond TTS outcome variability and inconsistent deployment across studies, each method carries inherent weaknesses that may undermine its ability to accurately capture subtle changes in postural control during landing. The RAW method is most commonly used with the vertical signal but is subject to random incidental peaks and has shown mostly ‘insufficient’ to ‘fair’ reliability [12]. The SA and TOP methods have demonstrated superior reliability; however, there are questions about how well these methods fit the underlying GRF data and about the large effect of trial length on the processed signal, with longer trials leading to a more horizontal signal and a longer TTS [9]. Additionally, the TOP method has only been applied to longer trial lengths (typically ≥ 20 s), which are not practical when testing large groups of athletes. Overall, studies comparing calculation methods within the same trials have produced TTS values ranging from 0.11 to 6.6 s [9] and have shown that no signal/threshold combinations produce a TTS value with clear optimum reliability [12]. These inconsistent outcomes and mixed reliability results are hindering research on DPS from advancing [13].

To address these limitations, recent research has explored different strategies including axis-specific method combinations [14], novel signal processing methods [15] and normalized stability thresholds [16,17]. No existing solution, however, has directly addressed the fundamental methodological limitations of the current approaches. An ideal TTS calculation method should incorporate formal statistical testing to accurately identify stable periods in the GRF signal, signal processing that preserves the underlying signal shape, statistically defined stability thresholds, acceptable reliability across all three GRF axes, and compatibility with shorter trial lengths for practical application in athlete monitoring contexts.

One approach with the potential to meet all of these requirements involves the use of the ‘two one-sided tests’ (TOST) equivalence testing method, a statistical approach used to determine whether two measurements can be considered practically the same within pre-defined limits. This approach differs from traditional null-hypothesis testing in that rather than testing whether observations differ significantly, the goal is to determine whether observed differences are small enough to be considered negligible [18]. In the context of calculating TTS, equivalence testing can be applied to windowed mean values to identify when the GRF signal becomes statistically indistinguishable from a stable baseline period representing acceptable limits of normal GRF variability. Specifically, using the TOST method, TTS may be represented by the first point in time where the confidence interval of the mean difference between the GRF signal and the stable reference period reaches and remains within the pre-defined equivalence margins [19].

Given the novelty of this approach, and the importance of defining and justifying the equivalence region when using the TOST method [20], an exploration of equivalence parameters and their effect on TTS outcomes is warranted. Therefore, the purpose of this study was to develop a novel method of calculating TTS using a TOST-based equivalence testing approach, developed and evaluated for the bilateral drop landing. The parameters of the TOST calculations were tuned using a structured grid search over plausible ranges in order to find the TOST equation that met pre-defined criteria for sensitivity, reliability and measurement error. The tuned TOST equation was then compared to existing calculation methods and assessed for agreement and reliability.

## 2. Materials and Methods

### 2.1. Participants

Twenty-six national team freestyle ski athletes (males = 14, females = 12; age = 22.36 ± 2.14 years; height = 1.75 ± 0.09 m; mass = 71.25 ± 11.76 kg) agreed to participate in this study. Using the Participant Classification Framework [21], participants were classified as either Tier 3—Highly Trained/National Level (*n* = 17) or Tier 4—Elite/International Level (*n* = 9). Data were collected at the start of the 2025–2026 season, as a part of routine testing to monitor performance on a variety of neuromuscular tasks. All participants were familiar with the assessment and were free of any injury at the time of data collection. Ethical approval was obtained from the University of Victoria’s Human Research Ethics Board (protocol number 23-0449-01), and all participants were explained the risks and benefits of participation in the study and provided informed consent prior to participation.

### 2.2. Procedures

Participants performed a double leg drop landing from a 50 cm box, placed 15 cm behind the rear edge of the force plates. Participants were asked to step straight out, leading with their preferred leg, and to land with one foot on each force plate, in a squat with thighs parallel to the ground and arms held directly in front of them. Each participant used their own preferred lead leg consistently across all five trials. Individual leg dominance was not recorded, and no asymmetry-based exclusion criterion was applied. Participants were instructed to “land soft and freeze like a statue”. During each trial, the landing position was held for six seconds, which was monitored by a metronome at 60 bpm and counted by the lead researcher. Following the completion of each trial, participants rested for 15 s. Each participant performed five landings.

GRF data were collected via a dual force plate setup (Advanced Medical Technologies Inc., Watertown, MA, USA) with GRFs in the V, AP and ML directions sampled at 1000 Hz and recorded by a custom LabVIEW software (Version 15.0, National Instruments, Austin, TX, USA). GRF signals from each plate were summed prior to any processing to yield a single bilateral signal for each axis, representing the net force governing whole-body stabilization. Data were filtered using a 2nd-order zero-lag low-pass Butterworth filter with a cut-off frequency of 40 Hz. Data were then cropped from time of impact (vertical GRF > 10 N) to 5 s post-impact, and the horizontal GRF signals (AP and ML) were rectified (converted to absolute values), which is consistent with standard practice [9]. All signals were normalized to body weight (BW) by dividing GRF(N)/BW(N) and are expressed as a percentage (%BW). All processing, calculations and analyses were conducted using R software (Version 4.4.2; Vienna, Austria).

### 2.3. Equivalence Testing (TOST)

Beginning from the time of impact, a centred rolling window of 50 ms duration (50 samples at 1000 Hz) was advanced one sample at a time, yielding mean and SD values at a temporal resolution of 1 ms for TTS detection. The 50 ms window duration was selected to provide sufficient samples for stable statistical estimates while remaining shorter than the timescale of automatic postural corrections of approximately 80–120 ms following a perturbation [22], reducing the likelihood that a single window spans a stabilization transition and thereby preserving sensitivity to the onset of stability. Next, the stable reference period was defined as the final 2 s of the trial (3–5 s post-impact), consistent with the reference period duration employed by Wright et al. [17]. The validity of this period as a stable reference was supported by reference period SD (SD_ref_) values (V: 0.59, AP: 0.24, ML: 0.18% BW) that closely matched those reported by Fransz et al. [12] (V: 0.53, AP: 0.23, ML: 0.27% BW), suggesting that the GRF signals had reached a level of low variability that is comparable to previous work. To verify that the reference period represented a settled state rather than an ongoing trend, a linear regression of GRFs on time was fitted within the reference window for each trial, and the proportion of reference period variance attributable to a linear trend was quantified. Subsequently, the mean difference for each window was calculated as the difference between each window mean and the mean of the reference period. A confidence interval was constructed around the mean difference using the Welch Standard Error and degrees of freedom with unequal n. The equivalence region representing acceptable GRF stability was defined by Delta, calculated as the equivalence scaling factor (k) multiplied by SD_ref_. Equivalence was declared whenever the two-sided confidence interval of the mean difference lay entirely within the equivalence margins. TTS was defined as the first window in a string of windows that remained equivalent to the reference period for a duration defined as dwell. k and dwell were identified as the parameters to be tuned in order to optimize for sensitivity, reliability and measurement error, using the criteria and procedure described in Section 2.4.1 and Section 2.4.3. k controls the strictness of the threshold for stability, and dwell controls the persistence requirement, both of which contribute to the definition of stability. The complete sequence of steps comprising a single TOST calculation is summarized in Table 1.

### 2.4. Data Analysis

#### 2.4.1. TOST Parameter Tuning

To quantify how TOST depends on the choice of k and the dwell time, a parameter sensitivity analysis was performed across a pre-defined grid of k (2–20) and dwell (100, 200, 300, 500, 750, 1000 ms). For each combination of k and dwell, TOST was computed for all available trials, resulting in 114 unique TOST scores per trial per GRF axis (19 k values × 6 dwell values). The distribution of TOST at each parameter setting was summarized by the median and interquartile range (IQR).

The local sensitivity of TOST to k was estimated using finite differences across adjacent k values within each dwell condition. Sensitivity was defined as the absolute change in the median TOST per unit change in k between successive grid points (|ΔTTS/Δk|), computed separately for each dwell time and GRF axis. The resulting sensitivity curves characterize regions where TOST was highly responsive to k versus regions of relative insensitivity to further increases in k. For each GRF axis and dwell condition, the lower bound of the acceptable parameter region was defined as the smallest k for which |ΔTTS/Δk| fell below the median sensitivity, computed as the median of |ΔTTS/Δk| across all k values (2–20), indicating that TOST had entered a region of relative insensitivity to the equivalence scaling factor. Parameter combinations that met this criterion were considered to yield behaviourally stable TOST estimates and were subsequently evaluated for reliability to identify preferred operating parameters.

#### 2.4.2. Reliability Analysis

Within-session trial-to-trial reliability was quantified using a two-way random-effects, absolute agreement, average measures intraclass correlation coefficient (ICC(2,5)) [23]. In this model, both athletes and repeated landings were treated as random effects, such that the reliability reflects the agreement of TOST across trials within an athlete. ICC values were computed separately for each axis × dwell × k combination and interpreted as ‘poor’ (<0.50), ‘moderate’ (0.50–0.75), ‘good’ (0.75–0.90) and ‘excellent’ (>0.90) [24]. Measurement precision was further quantified using the standard error of measurement (SEM), calculated for the averaged five-landing score as:

$$SEM={SD}_{mean}\times \sqrt{1-ICC(2,5)}$$

where SD_mean_ represents the between-athlete standard deviation of the averaged TOST score for the corresponding axis × dwell × k condition, such that SEM reflects the expected absolute measurement error of the averaged TOST value. The minimal detectable change at the 95% confidence level (MDC_95_) was calculated as:

$${MDC}_{95}=SEM\times 1.96\times \sqrt{2\;}$$

representing the smallest change in TOST that can be interpreted as exceeding the measurement error with 95% confidence. The ICC and SEM were summarized across k within the behaviourally stable parameter region by axis and dwell duration to determine whether the reliability remained consistent across stable parameter settings or continued to improve with increasing k.

#### 2.4.3. TTS Method Comparison

Prior to method comparison, a single k and dwell combination was selected for each GRF axis to yield one TOST value per trial for direct comparison against existing methods. Within the behaviourally stable parameter region, the lowest k value producing ‘good’ reliability (ICC ≥ 0.75) was selected with the aim of balancing reliability with threshold strictness, as lower k values impose a narrower equivalence region, preserving sensitivity to small fluctuations in the GRF signal. Where multiple dwell values met this criterion at a given k, the longest dwell was preferred, as a longer persistence requirement provides stronger evidence that the GRF signal had genuinely stabilized, consistent with the stability persistence criteria of 500–1000 ms employed in existing threshold-based methods [25,26]. For axes where no parameter combination achieved ICC ≥ 0.75, the combination yielding the highest observed ICC was selected, again preferring the longest dwell at equivalent ICC values.

**Figure 1:**
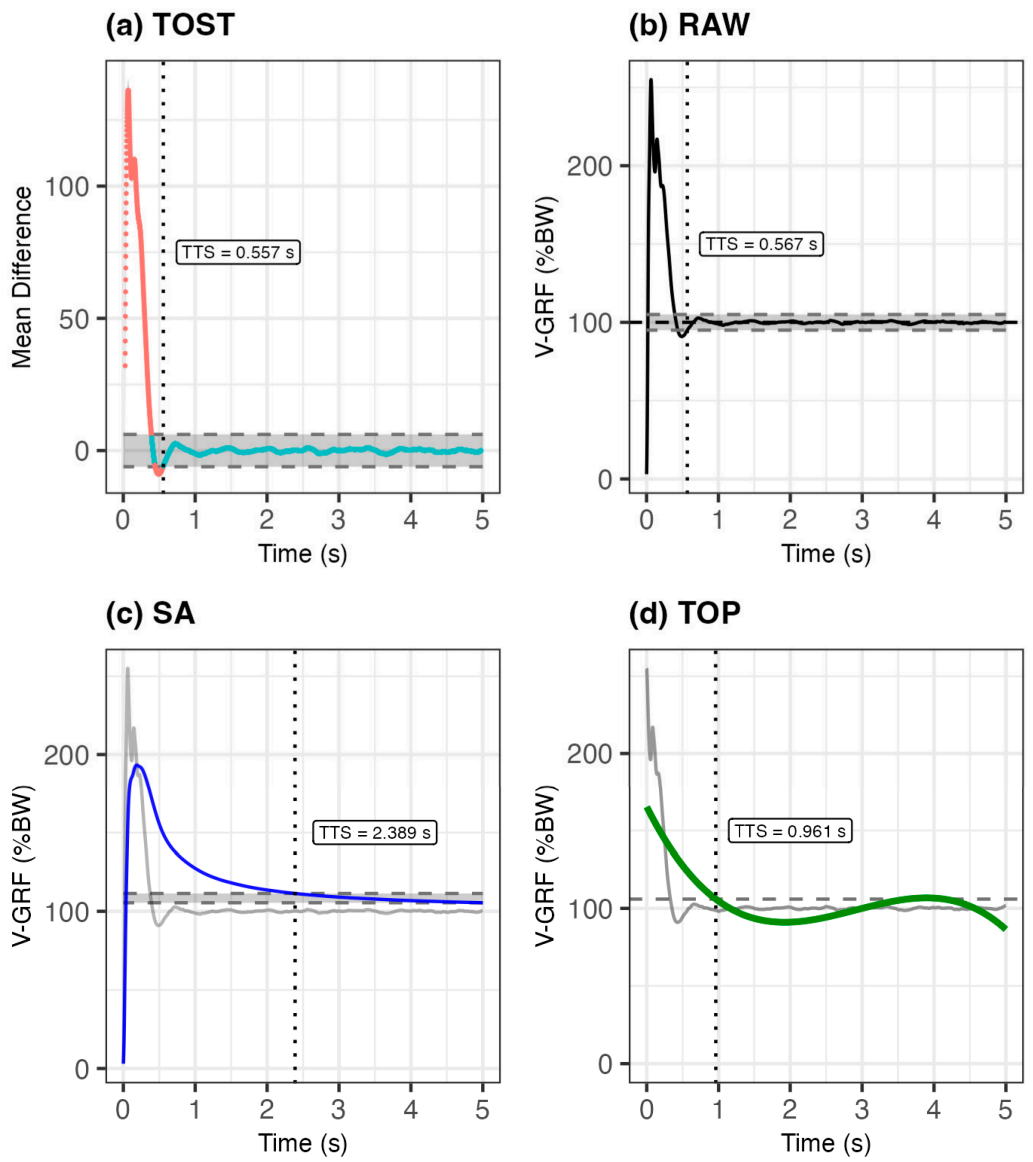
A visualization of the calculation of time to stabilization (TTS) using vertical ground reaction forces (V-GRFs) for a representative trial of a 50 cm drop landing. TOST panel displays mean difference vs. time (see items 1–4 of Table 1) with windows outside of the equivalence region coloured red and windows within the equivalence region coloured blue. All other panels display V-GRF vs. time. Horizontal dashed lines represent stability thresholds. Vertical dotted lines represent TTS. (**a**) TOST = two one-sided tests of equivalence method; (**b**) RAW = unprocessed method; (**c**) SA = sequential average method; (**d**) TOP = third-order polynomial method.

RAW, SA and TOP were calculated once for each GRF signal per landing trial. RAW was defined as the time taken for the unprocessed signal to remain within a pre-determined stability threshold expressed as a percentage of body weight: 5% BW for the vertical axis [27] and 1.057% BW/0.811% BW for the AP and ML axes, respectively, based on the methods employed by Brazen et al. [28]. SA was calculated by sequentially adding one data point at a time and determining when the resulting running average stabilized within 0.25 SD of the mean of the full signal [29]. For TOP, an unbounded third-order polynomial was fit to each signal using the function f(x) = a_0_ + a_1_x + a_2_x^2^ + a_3_x^3^, where a_3_ ≠ 0 [9], with TTS declared when the fitted signal reached axis-specific thresholds (V = 10 × SD_ref_, AP = 10 × SD_ref_, ML = 7 × SD_ref_) derived from the same stable reference period as TOST, ensuring comparable stability thresholds across methods [12]. Figure 1 provides a visualization of all TTS calculation methods used in this study.

Descriptive statistics (median and IQR) were calculated for each method and plotted for visual inspection. Reliability was quantified for each method using the ICC(2,5) and SEM as described in Section 2.4.2. The agreement between TOST and each existing method was assessed using Bland–Altman analysis, with bias quantified as the mean difference and limits of agreement defined as ± 1.96 SD of the differences. Proportional bias was assessed by regressing the mean difference on the mean of the two methods, with a statistically significant slope indicating that the agreement varied systematically with TTS magnitude.

## 3. Results

### 3.1. TOST Development and Optimization

#### 3.1.1. TOST Distribution

**Figure 2:**
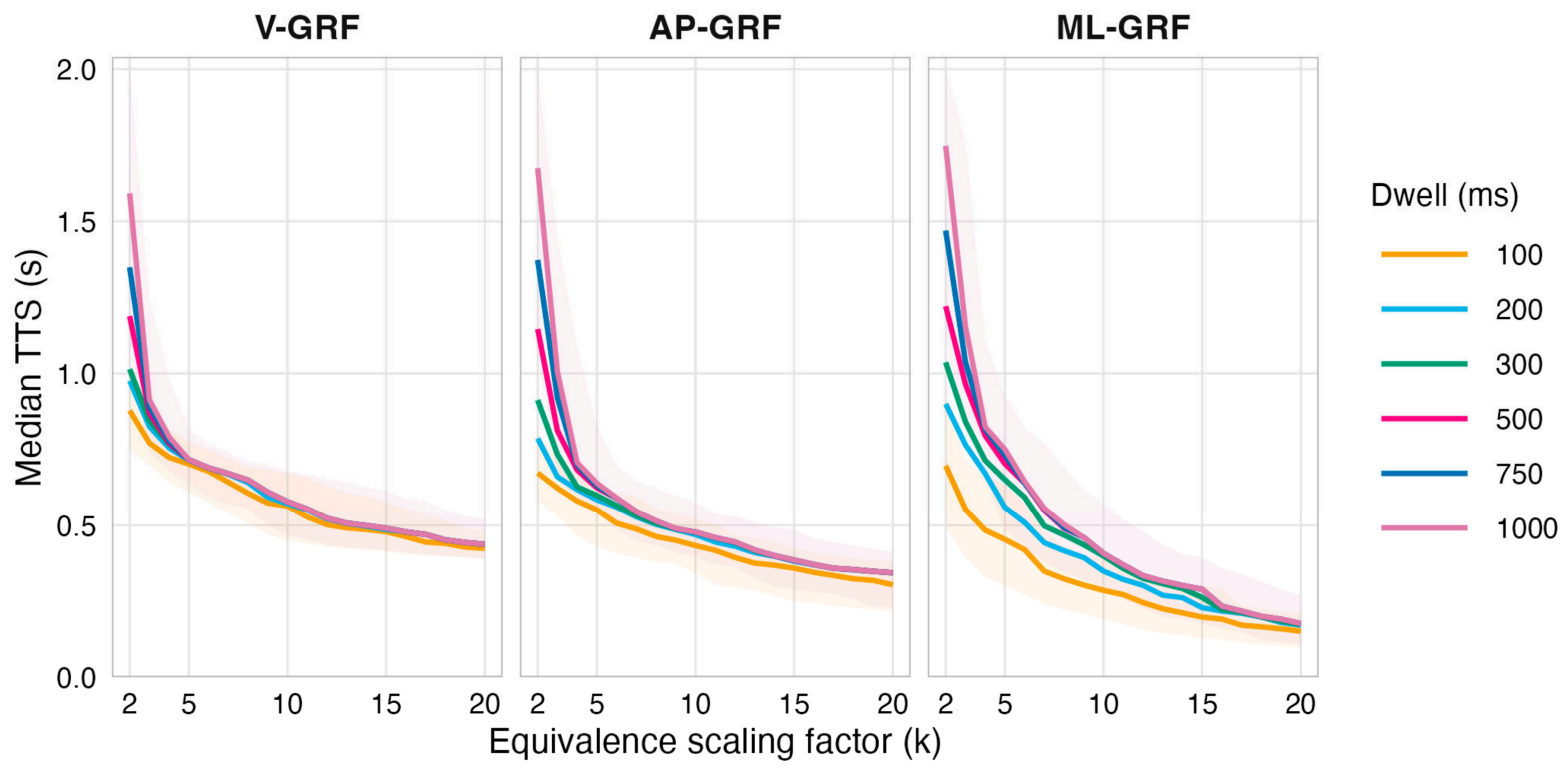
Median TTS values calculated via the TOST method (lines) with interquartile range (shaded ribbons, shown for dwell = 100 ms and dwell = 1000 ms only) as a function of equivalence scaling factor (k) and dwell duration across GRF axes.

The median TOST values with interquartile ranges across all axes and parameter combinations are presented in Figure 2. At the narrowest equivalence width (k = 2), the median TOST ranged from 0.67–1.75 s across axes and dwell conditions, reflecting high sensitivity to both parameters. TOST exhibited a nonlinear decline as k increased, with the steepest rate of change at low k values. By k = 20, the median TOST had converged to 0.15–0.44 s across axes, with markedly reduced variability. Dwell duration exerted its strongest influence at low k values, e.g., at k = 3, the difference in the median TOST between the shortest (100 ms) and longest (1000 ms) dwell conditions reached 0.38 s, 0.14 s and 0.60 s for the AP, V and ML axes, respectively. As k increased, this separation narrowed substantially, with dwell-related differences of less than 0.10 s observed across all axes by k = 15 and curves converging toward a stable range of TOST values.

#### 3.1.2. Parameter Sensitivity

**Figure 3:**
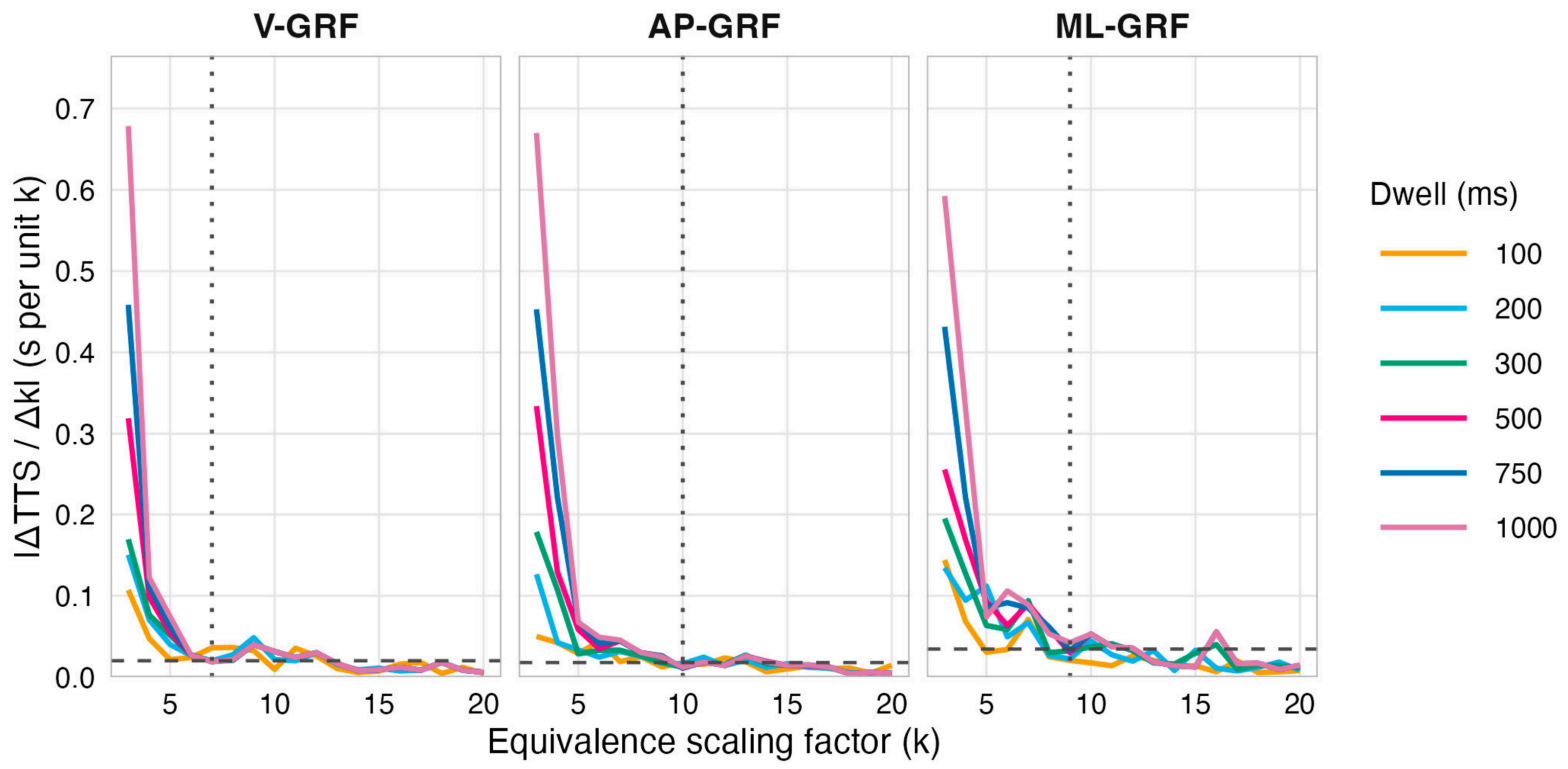
Absolute sensitivity of median TTS values calculated via the TOST method to equivalence scaling factor k (|ΔTTS/Δk|) across GRF axes and dwell conditions. Dashed horizontal line indicates the axis-specific median sensitivity threshold used to define the lower bound of the behaviourally stable parameter region. Dotted vertical line indicates the median stable k across dwell conditions (V-GRF: k = 7, AP-GRF: k = 10, ML-GRF: k = 9).

Sensitivity curves for all three GRF axes are presented in Figure 3. Across all axes and dwell conditions, TOST exhibited high sensitivity to k at low equivalence widths, with the largest changes in the median TOST per unit k observed at k = 3. At this point, the sensitivity ranged from 0.11–0.68, 0.05–0.67, and 0.13–0.59 s per unit k for the V, AP, and ML axes, respectively, with the spread across dwell conditions reflecting the strong influence of persistence requirements under strict equivalence criteria. The sensitivity decayed rapidly as k increased, with all axes demonstrating a region of relative insensitivity to further increases in k beyond a consistent threshold.

The axis-specific median sensitivity thresholds were 0.020, 0.018, and 0.035 s per unit k for the V, AP, and ML axes, respectively. The higher threshold for the ML axis reflects greater overall variability in that signal, consistent with previously reported difficulties in achieving reliable ML-GRF stabilization metrics. Based on these thresholds, the lower bounds of the behaviourally stable parameter region were k ≥ 7 (range: 7–10) for V-GRF, k ≥ 10 (range: 9–10) for AP-GRF, and k ≥ 9 (range: 8–11) for ML-GRF. At these stable k values, the sensitivity had fallen to a median of 0.019, 0.012, and 0.031 s per unit k for the V, AP, and ML axes, respectively, with the dwell-related spread reduced to less than 0.02 s per unit k across all axes.

The effect of dwell on sensitivity was pronounced at low k values but diminished markedly within the stable parameter region. For the V and AP axes, sensitivity curves for all dwell conditions had largely converged by their respective stable k thresholds. The ML axis demonstrated greater persistence of dwell-related differences across the stable region, with longer dwell durations maintaining slightly elevated sensitivity relative to shorter dwell conditions, consistent with the higher axis-level threshold for this signal. All combinations of dwell and axis-specific acceptable k-values were carried forward into reliability analyses.

#### 3.1.3. TOST Reliability Analysis

**Figure 4:**
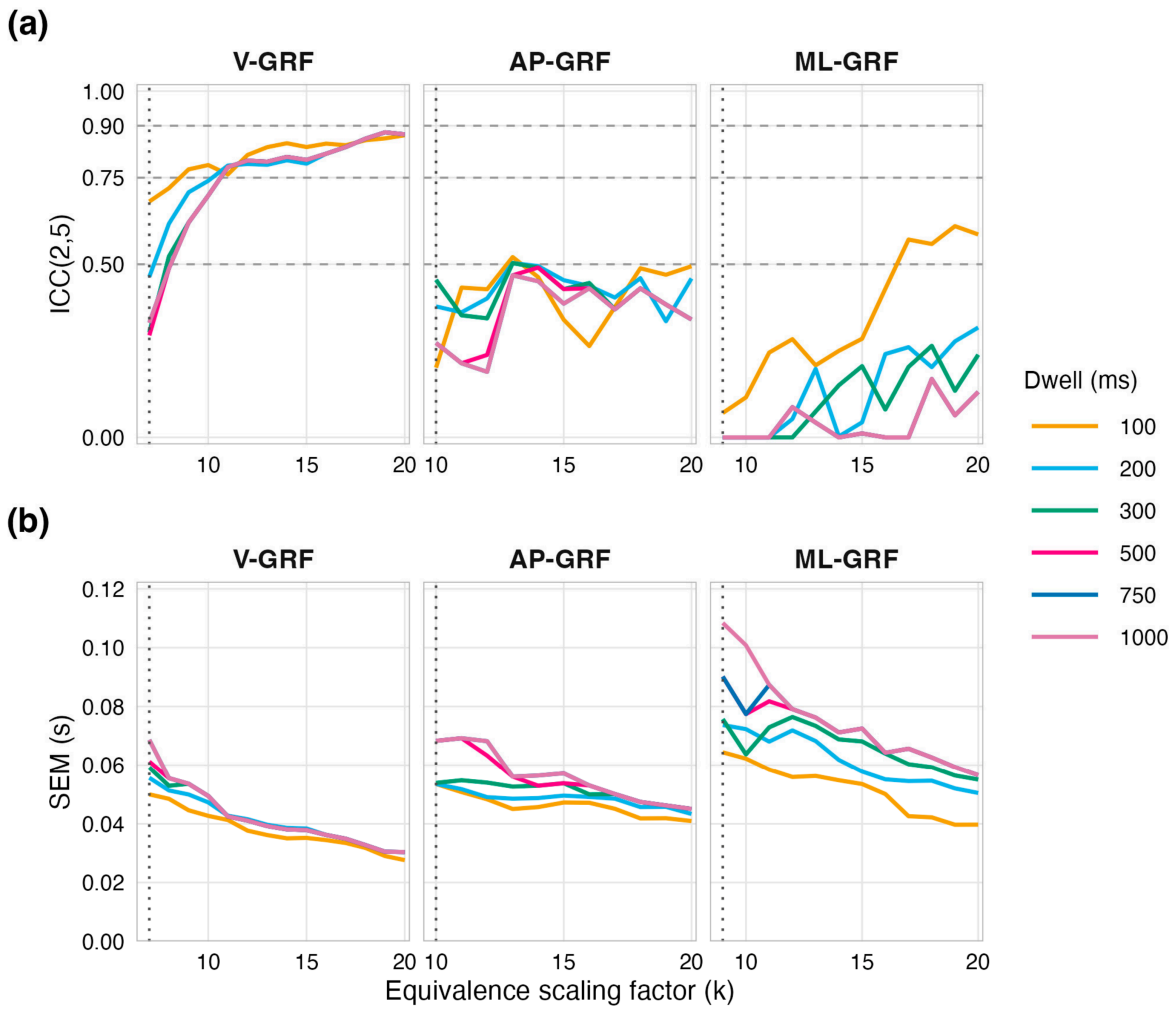
ICC(2,5) (**a**) and SEM (**b**) within the behaviourally stable parameter region for each GRF axis. Dashed horizontal lines in (**a**) indicate ICC classification thresholds (poor/moderate: 0.50, moderate/good: 0.75, good/excellent: 0.90). Dotted vertical lines indicate the axis-specific lower bound of the stable parameter region (V-GRF: k = 7, AP-GRF: k = 10, ML-GRF: k = 9).

The ICC(2,5) and SEM values within the behaviourally stable parameter regions are presented in Figure 4. The reliability differed substantially across GRF axes. For V-GRF, the ICC increased monotonically with k across all dwell conditions, ranging from 0.287 at k = 7 to 0.881 at k = 20. Good reliability (ICC ≥ 0.75) was achieved consistently across all dwell conditions from k = 11 onwards, with dwell exerting only a modest effect on the ICC throughout the stable region (median ICC: 0.789–0.838 across dwell conditions). The SEM values were low throughout, ranging from 0.028–0.069 s and decreasing with increasing k. AP-GRF demonstrated ‘poor’ to ‘moderate’ reliability throughout the stable parameter region (ICC = 0.189–0.518), with no parameter combination achieving ICC ≥ 0.75. The ICC was highest at k = 13 (maximum ICC = 0.518) with no clear monotonic trend. SEM decreased with increasing k (0.069 s at k = 10 to 0.041 s at higher k values), with longer dwell durations associated with modestly higher SEMs. ML-GRF demonstrated the poorest and most variable reliability, with ICC ranging from −0.966 to 0.606 across the stable region. Negative ICC values occurred at lower k under longer dwell conditions, reflecting within-athlete trial-to-trial variability exceeding between-athlete variability under these parameter settings. Dwell exerted a pronounced negative effect, with the median ICC declining from 0.269 at dwell = 100 ms to −0.021 at dwell ≥ 500 ms. SEM increased markedly with dwell duration, from 0.040–0.055 s at dwell = 100 ms to 0.073–0.115 s at dwell ≥ 500 ms.

Based on the selection criteria described in Section 2.4.3, axis-specific parameters were identified for method comparison (Table 2). For V-GRF, k = 11 with dwell = 1000 ms was selected as the lowest k at which good reliability was achieved consistently across all dwell conditions (ICC(2,5) = 0.781 [95% CI: 0.613, 0.890], SEM = 0.043 s). For AP-GRF, no parameter combination achieved ICC ≥ 0.75; k = 13 with dwell = 100 ms was therefore selected based on the highest observed ICC (ICC(2,5) = 0.518 [95% CI: 0.146, 0.759], SEM = 0.045 s). Similarly for ML-GRF, k = 19 with dwell = 100 ms was selected (ICC(2,5) = 0.606 [95% CI: 0.297, 0.803], SEM = 0.040 s), representing the highest reliability achievable within the stable parameter region. The preference for a shorter dwell in AP and ML reflects the systematic deterioration of reliability with longer persistence requirements observed in these axes.

### 3.2. TTS Method Comparison

#### 3.2.1. Descriptive Statistics

**Figure 5:**
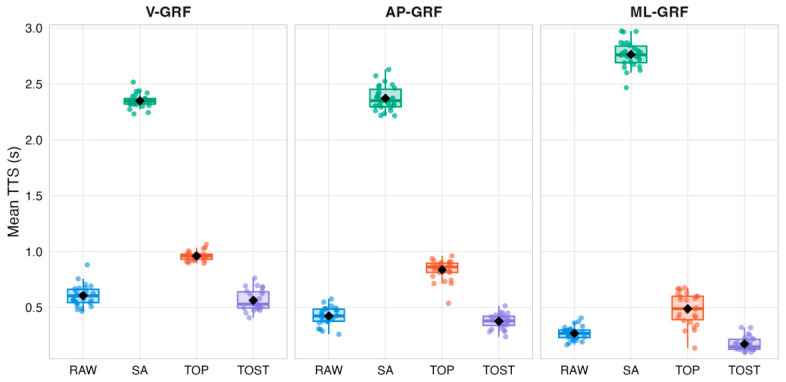
Distributions of athlete-level mean time to stabilization (TTS) for each calculation method (RAW, SA, TOP, TOST) across the three ground reaction force axes (V-GRF, AP-GRF, ML-GRF). Each coloured point represents one athlete’s mean TTS averaged across their five landings. The boxplot shows the interquartile range (25th to 75th percentile) with the horizontal line indicating the median, and the whiskers extending to 1.5 times the interquartile range. The black diamond indicates the group mean across athletes.

Descriptive statistics for all four methods are presented in Figure 5. TOST, RAW, and TOP produced broadly comparable distributions for V-GRF, with median athlete-level TTS values of 0.53 s, 0.61 s, and 0.96 s, respectively. TOST and RAW were most similar in the vertical axis, while TOP produced consistently longer estimates. For AP-GRF, TOST (median = 0.38 s) and RAW (median = 0.43 s) again showed close agreement, with TOP producing substantially longer estimates (median = 0.86 s). The ML axis showed the greatest separation among the three non-SA methods, with TOST producing the shortest estimates (median = 0.15 s), followed by RAW (median = 0.27 s) and TOP (median = 0.49 s), and the widest variability was observed for TOP (IQR = 0.39–0.60 s). Across all axes, SA produced markedly higher TTS values than all other methods, with median athlete-level TTS values of 2.35 s, 2.35 s, and 2.76 s for V, AP, and ML, respectively, and narrow interquartile ranges, consistent with the strong influence of trial length on the SA processing method.

#### 3.2.2. Method Reliability Comparison

**Figure 6:**
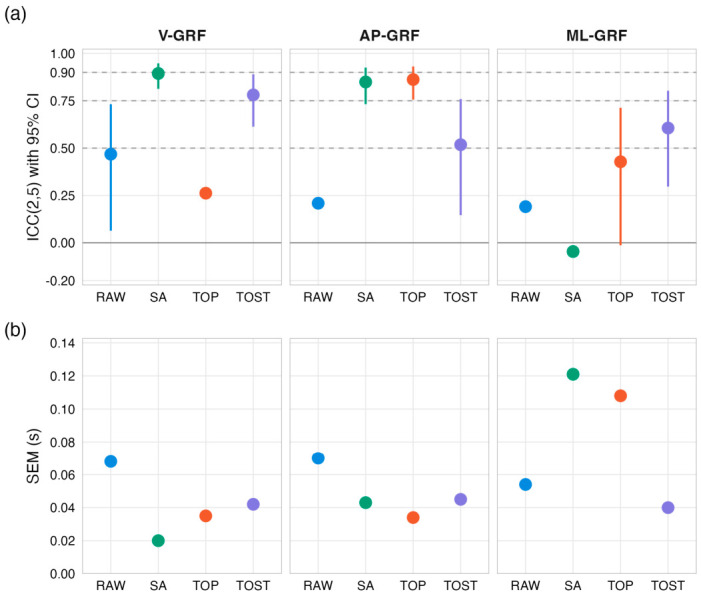
ICC(2,5) with 95% confidence intervals (**a**) and SEM (**b**) for each calculation method across GRF axes. Dashed horizontal lines in (**a**) indicate ICC classification thresholds (poor/moderate: 0.50, moderate/good: 0.75, good/excellent: 0.90).

The reliability results for all methods across GRF axes are presented in Table 3 and Figure 6. For V-GRF, TOST demonstrated good reliability, outperforming RAW and TOP. SA achieved excellent reliability in the vertical axis; however, this was accompanied by a within-athlete CV of only 1.7%, suggesting that the SA values were dominated by trial length rather than genuine between-trial variability in postural control. For AP-GRF, SA and TOP demonstrated the highest reliability, while TOST achieved moderate reliability and RAW performed poorly. The high CVs for RAW (36.8%) and TOST (25.7%) in the AP axis reflect the greater trial-to-trial variability inherent in horizontal GRF signals. For ML-GRF, TOST produced the highest reliability of all methods and the lowest SEM, despite only reaching moderate classification. RAW and TOP both performed poorly, while SA produced a negative ICC, indicating that the within-athlete trial-to-trial variability exceeded the between-athlete variability, consistent with the known sensitivity of SA to trial length artefacts in the horizontal axes.

#### 3.2.3. Bland–Altman Analysis

**Figure 7:**
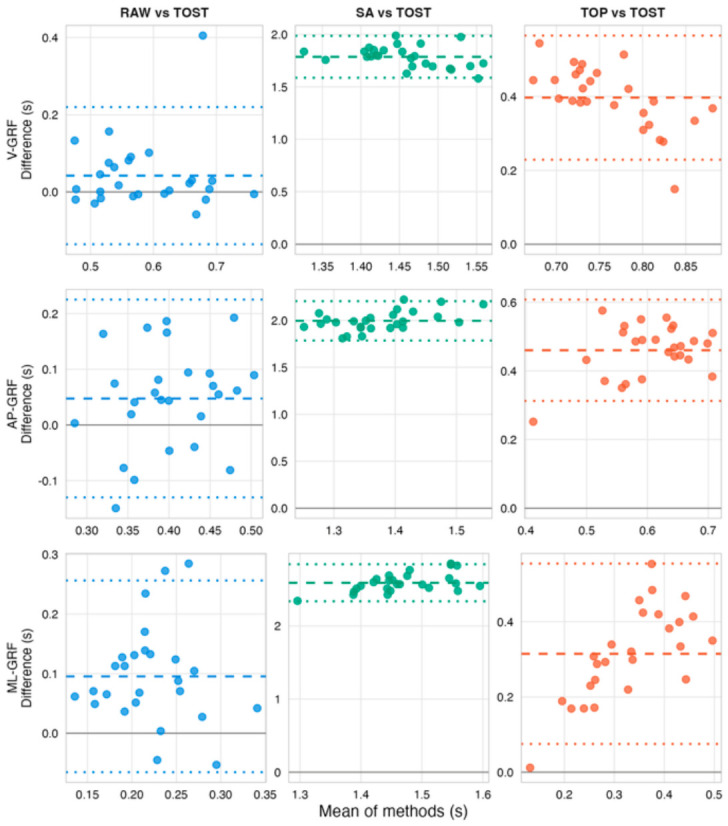
Bland–Altman plots comparing each traditional TTS calculation method against TOST across GRF axes. Each point represents one athlete’s mean TTS across five landings. Dashed lines indicate bias (mean difference); dotted lines indicate 95% limits of agreement. Note that axes are scaled independently within each panel.

The agreement between TOST and existing methods is presented in Figure 7. TOST showed close agreement with RAW across all three GRF axes, with small positive biases of 0.042 s, 0.048 s, and 0.095 s for V, AP, and ML, respectively, indicating that RAW produced slightly higher TTS estimates than TOST in all axes. The limits of agreement were comparable across axes (LoA width: 0.32–0.36 s), and no significant proportional bias was detected (r = 0.028, 0.197, −0.053; all *p* > 0.33), indicating that the agreement between RAW and TOST was consistent across the range of TTS values. SA showed large systematic bias relative to TOST in all axes (bias: 1.79 s, 1.99 s, and 2.59 s for V, AP, and ML, respectively), with limits of agreement spanning approximately 0.40–0.51 s around this bias. Significant proportional bias was detected for SA in AP (r = 0.507, *p* = 0.008) and ML (r = 0.556, *p* = 0.003), and it approached significance in V (r = −0.384, *p* = 0.053), indicating that the overestimation by SA relative to TOST increased systematically with the TTS magnitude in the horizontal axes. TOP consistently overestimated TTS relative to TOST across all axes (bias: 0.397 s, 0.460 s, and 0.315 s for V, AP, and ML). Significant proportional bias was detected for TOP in V (r = −0.681, *p* < 0.001) and ML (r = 0.728, *p* < 0.001), indicating that the magnitude of disagreement between TOP and TOST varied systematically with TTS. In the vertical axis, larger TTS values were associated with smaller differences between methods, while in ML, the opposite pattern was observed.

## 4. Discussion

This study proposes a novel method for calculating TTS that employs equivalence testing to identify when post-landing GRF signals become statistically indistinguishable from a stable reference period. A structured parameter tuning framework integrating sensitivity, reliability, and measurement error analysis yielded axis-specific parameter recommendations. For V-GRF, equivalence scaling factors of k ≥ 11 yielded behaviourally stable TOST estimates with consistently ‘good’ reliability (ICC(2,5) ≈ 0.76–0.88) and low SEM (0.035–0.038 s), regardless of the dwell value. AP and ML-GRF demonstrated low sensitivity to parameters at k ≥ 10 and 9, respectively, although the reliability was moderate at best and dependent on dwell conditions. TOST yielded close agreement with the RAW method with small consistent bias and superior reliability across all three axes. SA achieved high ICCs in the V and AP axes but with large systematic bias and very narrow score distributions, indicating the strong influence of trial length on SA estimates. TOP showed improved reliability in the AP axis but demonstrated significant proportional bias relative to TOST in V and ML.

### 4.1. TOST Development and Optimization

Variability and inconsistency in TTS outcomes arising from differences in calculation methods used across studies are well documented [9] and point to the need for clearly defined and systematically tuned parameters when developing these methods. Equivalence testing requires careful consideration when defining the equivalence region, as equivalence can be concluded firmly only when compared with a reference value of well-established efficacy [19]. Similarly, TTS outcomes are highly dependent on the stability threshold used [12]. By systematically testing the effect of the equivalence width and persistence on TTS outcome, this study has identified parameter combinations that yield behaviourally stable TTS outcomes and has reported the associated reliability and measurement error of these calculations.

The tuning of the equivalence region is comparable to previous TTS methodological studies that have explored the effect of stabilization thresholds using multiples of body weight or of GRF standard deviation from a presumed stable window and have similarly shown that TTS decreases in an exponential manner as the stability threshold is loosened [12]. It is clear that no matter what calculation strategy is employed, the TTS values are highly dependent on the stability threshold used, particularly at stricter threshold levels. In addition to exploring the effect of the equivalence width, the current approach also necessitated an examination of the duration for which the signal remained stable. Some previous TTS calculation methods using a signal-to-threshold approach necessitate that the signal remains within the threshold for a given period of time (e.g., 0.5 s) since the signal may intersect the threshold on multiple occasions [25,26]. Other methods, such as SA and TOP, converge by definition towards an asymptote and therefore will cross the stability criterion only once [29,30]. The TOST processed signal preserves the shape of the raw GRF signal, and thus, a sufficient dwell period is required to avoid falsely declaring stability while the signal continues to fluctuate. Theoretically, shorter dwell values increase the risk of prematurely declaring stability, while longer dwell values risk capturing variation in static postural stability or task non-compliance rather than the dynamic-to-stable transition of interest.

Beyond stable k thresholds of 7, 10, and 9 for V, AP and ML-GRF, respectively, the TOST values became sufficiently insensitive to further changes in k or dwell, such that the calculated TTS values were no longer sensitive to arbitrary threshold decisions and can be interpreted with greater confidence as reflecting genuine differences in postural control rather than methodological artefact. Within these stable parameter regions, the ICC trended upward with increasing k for V-GRF across all dwell values, reaching ‘good’ reliability (ICC ≥ 0.75) from k = 11 onwards with a trivial dwell effect. SEM decreased with increasing k, regardless of dwell, reaching 0.043 s at k = 11 and dwell ≥ 300 ms. The reliability for AP and ML was ‘moderate’ at best and dependent on the dwell conditions, with shorter dwell values producing clearly better ICC and SEM. Previous studies have similarly assessed the reliability of different TTS calculation methods as a prerequisite for accurate estimation of sensorimotor function. Fransz et al. [12] reported that the RAW and RMS methods showed ‘insufficient’ to ‘fair’ reliability regardless of threshold selection, while TOP showed ‘moderate’ reliability for V and AP at thresholds around 10 SD. The current finding of ICC > 0.75 with k ≥ 11 in the vertical axis is broadly consistent with these results, given that k similarly represents a multiple of the reference period SD, scaling SD_ref_ to define the equivalence region (Delta). For AP, maximum ICC values of 0.521 using TOST are comparable to, but lower than, previous values reported for TOP [12]. TOST also showed only moderate reliability in the ML axis, consistent with previous findings [12,15]. It is possible that the landing task used in the current study, which involved a primarily vertical drop with minimal forward projection, did not sufficiently challenge stability in the horizontal axes. Regarding measurement error, the SEM values associated with TOST in the current study were substantially lower than in previous work. García-Massó et al. [15] reported SEM values of approximately 0.3–0.5 s for the RAW and RMS methods and 0.1–0.5 s for SA and TOP. Although methodological differences make direct comparison difficult, even the maximum SEM values in the present study are considerably smaller, pointing to the potential of TOST as a methodology with lower measurement error and therefore a greater ability to detect small differences in TTS attributable to injury or training status.

Previous efforts to determine optimal TTS parameters based solely on reliability have yielded mixed results, with no combination of existing signal processing and threshold combinations producing consistently optimal reliability [12]. In the current study, a clear zone of behavioural stability, ‘good’ reliability and low measurement error was demonstrated for V-GRF using TOST. The behaviourally stable parameter region was identified from the signal sensitivity criterion prior to reliability analysis and therefore reflects the point at which TTS becomes insensitive to further threshold widening, a property of the GRF signal structure rather than a sample-specific reliability artefact. For parameter selection, the lowest k value and longest dwell producing ‘good’ reliability were preferred over the combination yielding the highest ICC, balancing reliability with threshold strictness and avoiding the risk of missing meaningful signal variability through an overly wide equivalence region. For AP and ML, where no parameter combination achieved good reliability, the highest observed ICC was selected. Notably, at the selected parameter values, the sensitivity had fallen below the SEM for all three axes, indicating that the uncertainty in threshold selection had become smaller than the inherent measurement error of TOST. This provides a practically grounded interpretation of behavioural stability based on the point at which further refinement of k would produce changes in TTS smaller than the measurement precision of the instrument itself. Although the reliability in AP and ML was not substantially improved relative to previous methods, the combination of behaviourally stable estimates, low SEM, and the methodological advantages discussed below makes TOST a compelling candidate for further investigation in the horizontal axes.

### 4.2. TOST Method Comparison

Given the lack of a gold standard TTS measurement for quantifying DPS as the transition from a dynamic to a stable state following a drop landing, TOST is best evaluated by direct comparison with the most common established techniques. A primary finding of this study was that TOST showed high agreement with the RAW method in the vertical axis while displaying improved within-session reliability. In a previous review, the RAW method was the most commonly used approach for measuring TTS in the vertical axis (67%; 29/43) compared to SA (26%; 11/43) and TOP (7%; 3/43) [2]. Furthermore, most widely used commercial force platform systems report TTS only in the vertical axis using the RAW method. Based on the current findings, TOST appears to be a suitable replacement for RAW in the vertical axis, providing similar estimates of dynamic postural control while improving reliability and reducing measurement error. TOST also displayed close agreement with small consistent bias and superior reliability in comparison to RAW in the AP and ML axes, although it should be noted that RAW is not commonly used to assess TTS in these axes, and studies that have done so have not provided detailed methodological justification [28,31]. Researchers and practitioners should consider replacing RAW with TOST when measuring TTS from vertical GRFs.

In comparison to TOP, TOST produced consistently lower TTS scores across all axes with significant proportional bias detected in V and ML, indicating that the magnitude of disagreement varied systematically with TTS magnitude. TOST was substantially more reliable in the vertical axis, while TOP displayed better reliability for AP. The ICC values reported for TOP in the current study were lower than those previously reported for the vertical axis but were higher for AP [12], a pattern that may reflect the shorter trial length used here, as TOP is typically applied to trials of 20 s or longer and may not be well-suited to the 5 s trials used in the present study. Interestingly, the significant proportional bias observed in the vertical axis was negative, indicating that TOP and TOST converged at higher TTS values. This suggests that in populations characterized by longer stabilization times, the two methods may produce more comparable estimates. Regardless, the systematic disagreement across the TTS range means that TOP and TOST cannot be used interchangeably, and TOP is likely not suitable for measuring TTS in the vertical axis using 5 s trial lengths.

In this study, SA was a clear outlier, producing TTS estimates on an entirely different scale from all other methods. SA showed large systematic bias relative to TOST across all axes, with significant proportional bias in AP and ML. Previous research has identified mechanistic limitations of the SA method, including that the cumulative average is prone to producing lower TTS scores when force oscillations are increased and that using SD values derived from the entire landing signal overestimates the stability threshold [32]. As expected, based on prior research [29], SA produced strong ICC values in the vertical and AP axes. Considering that SA produced values on a different scale from all other methods, showed magnitude-dependent bias in the Bland–Altman analysis, and has been shown to carry the largest trial length effect of any TTS method (>240%) [8], these findings indicate that SA values were governed substantially by trial length rather than by genuine differences in postural control. In the ML axis, SA produced a negative ICC, indicating that within-athlete trial-to-trial variability exceeded between-athlete variability. Although some previous studies have reported adequate ML reliability for SA [12,29], this was not replicated in the current study, likely because the double-leg vertical drop landing task did not sufficiently challenge participants in the frontal plane. Across all axes, TOST cannot be used interchangeably with SA, and the use of SA as a measure of dynamic postural stability should be questioned given the strong evidence of trial length dependence.

It is also notable that TOST achieved the highest reliability of all methods in the ML axis, despite only reaching moderate classification. RAW, TOP, and SA all performed poorly in ML, consistent with some previous reports [12,15]. That TOST leads on ML reliability, even in a task that likely did not optimally challenge frontal plane stability, suggests further promise for this method in the horizontal axes, particularly if applied to tasks with greater medio-lateral demand.

### 4.3. Methodological Considerations and Practical Implications

**Figure 8:**
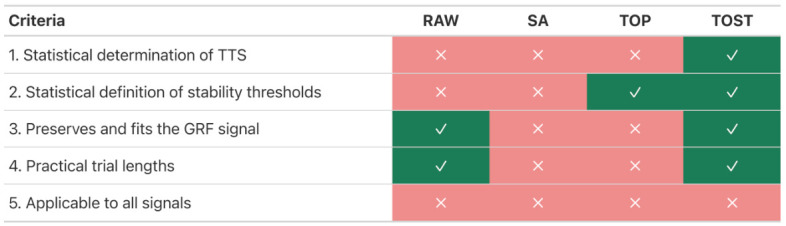
Summary of ideal TTS calculation method characteristics. Green check-marks denote when each given criteria is met for each individual calculation method.

Beyond reliability and agreement, TOST offers several methodological and practical advantages over existing approaches, which are summarized in Figure 8. TOST is the first TTS calculation method to use formal statistical testing to define stability, identifying the earliest time point at which the GRF signal is statistically equivalent to a stable reference period. The two one-sided tests calculation was selected as an appropriate statistical framework as it provides empirical evidence of equivalence between two measurements rather than simply failing to reject a null hypothesis of difference [20]. In this implementation, stability is defined as the earliest time point at which the GRF signal remains statistically equivalent to a reference distribution derived from the end of the trial. In contrast, RAW relies on an arbitrary fixed threshold, while SA and TOP depend on a fitted signal converging toward an asymptote. TOST provides a probabilistic rather than deterministic estimation of postural control by constraining the uncertainty of the GRF signal, requiring both the mean difference and its confidence interval to fall within the equivalence region and by further requiring that this condition persist for a defined dwell duration.

The statistical rigor of this approach also extends to the definition of the stability threshold. RAW has primarily used an arbitrary threshold of ±5% body weight, while SA uses the mean and SD of the entire landing signal, which includes large impact forces and thereby overestimates the stability threshold. TOP thresholds are often more robustly defined using a low-variability period near the end of the trial, and the current TOST approach similarly uses descriptive statistics from a stable reference period, but rather than adopting a fixed SD multiple, the width of the equivalence region (Delta = k × SD_ref_) is set by tuning the scaling factor k, together with the dwell duration, through the structured analysis described in Section 2.4. The final two seconds of each trial were selected as the reference period based on previous research [17], and the validity of this choice was supported by the reference period SD values closely matching those reported by Fransz et al. [12] across all three axes, suggesting that the five-second trial length was sufficient to capture a state of low variability in the GRF signal. A linear-trend analysis further supported this, with the systematic trend accounting for a median of 1.5%, 2.7%, and 5.7% of the reference period variance in the V, AP, and ML axes, respectively, indicating that the reference period variability was predominantly non-directional fluctuation about a settled state rather than ongoing drift. Future research may seek to further develop objective criteria for defining periods of stable posture within a landing task trial, including the use of normalized or externally derived reference periods that reduce dependence on the within-trial signal [16].

A further advantage of TOST lies in its fidelity to the underlying GRF signal. The TOST processing approach, which computes windowed means and confidence intervals relative to a stable reference, preserves the shape of the raw GRF signal while providing a statistically grounded representation of signal uncertainty. This addresses a key limitation of SA and TOP, which is that fitted signals may not accurately capture small but meaningful fluctuations in GRFs during the transition to a stable state following landing [14]. Additionally of note, although TOST is statistically more elaborate than RAW, this complexity is contained within the calculation itself; as a single-pass, deterministic algorithm requiring no manual fitting or subjective threshold selection, it is no more demanding to apply in practice than existing methods once implemented.

Another practical advantage of TOST is its independence from trial length. Prior research has quantified the large effect of trial length on SA and TOP outcomes, questioning the validity of these methods on the grounds that maintaining a stable position for longer should not logically produce a higher TTS value [9]. Additionally, the TOP method in particular appears to require trial lengths of at least 20 s, which are impractical for applied testing batteries, where the drop landing is one of several tests and multiple trials with rest intervals are required per participant. Unlike SA and TOP, TOST is not affected by trial length provided the trial is sufficiently long to capture a stable reference period. This allows trial length to be adapted to the population (e.g., elite vs. recreational athletes, healthy vs. injured athletes) and task (e.g., single- vs. double-leg, increased drop height) of interest and permits shorter trials that make large-group testing more practical. In this study, a five-second trial length was selected, as trials of greater duration were considered impractical and likely to introduce fatigue, given that the landing position required sustained active muscular contraction in a parallel squat, which we hypothesize could increase variability in the reference signal, though this remains to be tested.

Finally, in line with previous research, this study did not provide unequivocal support for applying the same TTS calculation method across all three GRF signals [15], as TOST achieved only ‘moderate’ reliability under specific parameter conditions in AP and ML. Nevertheless, TOST yielded behaviourally stable estimates in these axes, and the pattern of TTS response to increasing k is consistent with previous findings [12,15]. Notably, TOST achieved the lowest SEM and highest ICC of all methods in the ML axis, despite the use of a landing task that did not sufficiently challenge frontal plane stability. These results suggest that the method may hold potential for horizontal axis assessment when applied to tasks with greater AP and ML demand, but the present findings should not be interpreted as validated outcomes for this task. Consistent with this, directional dynamic stability has been shown to depend on the plane of the landing task, with frontal-plane stability deficits emerging specifically during lateral rather than forward landings [33], reinforcing the expectation that tasks imposing greater medio-lateral demand are required to meaningfully challenge and assess ML axis stability. Until improved reliability can be demonstrated for AP and ML, it is recommended that practitioners prioritize TOST in the vertical axis, where the evidence base is strongest. Future research should examine TOST applied to landing tasks that specifically load the horizontal axes, such as lateral jump landings to challenge the ML component and forward or backward landings to challenge the AP component, performed either bilaterally or unilaterally. Between-session reliability and criterion validity, evaluated against clinical, functional, and performance measures of dynamic postural control, should also be established.

### 4.4. Limitations

Several limitations of the present study should be acknowledged. First, reliability was assessed within a single testing session, reflecting trial-to-trial consistency rather than between-session reproducibility. Between-session reliability is a critical prerequisite for the use of TOST in longitudinal monitoring and return-to-sport assessment and should be examined in future research. Second, participants were elite athletes experienced in drop landing, which may limit the generalizability of the parameter recommendations to recreational or injured populations where GRF signal characteristics and postural control strategies may differ. External validation of the recommended k and dwell values across populations, landing tasks, and force plate systems is therefore an important prerequisite before these parameters can be adopted broadly. Third, the bilateral double-leg drop landing task used in this study involved primarily vertical force production with minimal horizontal plane challenge, which likely contributed to the poor reliability observed in the AP and ML axes. A task with greater sagittal or frontal plane demand may yield more favorable results for horizontal axis TOST. Fourth, the number of trials was set at five based on convention rather than optimization, and future research should determine the minimum number of trials required to achieve stable averaged TOST estimates. Finally, while the reference period was validated against published SD values, the definition of a true stable state remains an open question in the TTS literature, and future work should seek to establish objective criteria for confirming postural stability within a landing trial.

### 4.5. Conclusions

This study introduced TOST, a novel equivalence testing-based approach to calculating TTS from force plate-derived GRFs following a drop landing. A structured parameter tuning framework identified axis-specific equivalence scaling factors and persistence requirements that yielded behaviourally stable TTS estimates, with ‘good’ reliability and low measurement error demonstrated for the vertical axis. In this axis, TOST showed close agreement and superior reliability when compared with the RAW method, alongside substantially lower measurement error than that previously reported for any existing method, and it is therefore recommended as a replacement for RAW in the vertical axis. The reliability in the AP and ML axes, by contrast, was only poor to moderate, indicating that horizontal axis application will require tasks with greater horizontal demand and further validation. Among the remaining methods, the markedly different scale of the SA values and their magnitude-dependent bias relative to TOST, considered alongside prior work that directly manipulated trial length [9], suggest that SA is substantially influenced by trial length rather than postural control, while TOP demonstrated systematic proportional bias that varied with TTS magnitude. Collectively, these findings support TOST as a statistically principled and practically viable alternative to existing threshold-based TTS definitions.

TOST addresses several longstanding methodological limitations of existing approaches: it employs formal statistical equivalence testing rather than arbitrary threshold crossing, defines stability thresholds using statistical principles, preserves the shape of the raw GRF signal, and is not sensitive to trial length, enabling shorter, more practical protocols suited to frequent monitoring of large athlete groups. The reliability in the AP and ML axes remains a challenge shared by all existing methods, and future research should examine TOST in landing tasks with greater horizontal plane demand and evaluate between-session reproducibility. By introducing equivalence testing to the calculation of TTS and tuning its parameters through a structured sensitivity and reliability analysis, building on prior methodological work that systematically examined thresholds and reliability [9,12], TOST provides a transparent and reproducible foundation for future investigation of dynamic postural control in athletic populations.

## Figures and Tables

**Table 1:** Ordered specification of the time to stabilization (TTS) two one-sided tests (TOST) calculation. ms = milliseconds; SD = standard deviation; SD_ref_ = standard deviation of the reference period; CI = confidence interval; k = equivalence scaling factor.

Step	Component	Definition
**Signal windowing**		
1	Rolling window	Fixed 50 ms centred window, advanced one sample at a time from time of impact
2	Windowed statistics	Mean and SD computed within each window, giving 1 ms temporal resolution
**Reference comparison**		
3	Reference period	3–5 s post-impact, providing the stable comparison distribution
4	Mean difference	Difference between each window mean and the reference period mean
5	Confidence interval	Two-sided CI of the mean difference (Welch standard error and degrees of freedom, unequal *n*)
**Equivalence and stability**		
6	Equivalence region	Delta = k × SD_ref_, where k is the equivalence scaling factor and SD_ref_ the reference period SD
7	Equivalence test	Equivalence declared when the CI of the mean difference lies entirely within ±Delta
8	Persistence (dwell)	Equivalence must be maintained continuously for the dwell duration
9	TOST	First time point of the first window in a dwell-length run of continuously equivalent windows

**Table 2:** Summary of TOST parameters to be used for comparison against existing methods.

	V-GRF	AP-GRF	ML-GRF
**Selected parameters ^1^**			
k	11	13	19
Dwell (ms)	1000	100	100
**Results at selected parameters**			
Median TTS (s)	0.53	0.38	0.15
Sensitivity (|ΔTTS/Δk|)	0.019	0.012	0.031
ICC(2,5) [95% CI]	0.781 [0.613, 0.890]	0.518 [0.146, 0.759]	0.606 [0.297, 0.803]
SEM (s)	0.043	0.045	0.040

^1^ Parameters were selected as the lowest k achieving ICC ≥ 0.75 across all dwell conditions (V-GRF), or the highest observed ICC where this threshold was not met (AP-GRF, ML-GRF). The longest dwell was preferred at equivalent ICC.

**Table 3:** Summary of reliability data for each calculation method across GRF axes.

	ICC(2,5) [95% CI] ^1^	SEM (s)	MDC_95_ (s)	CV (%)
**V-GRF**				
RAW	0.468 [0.064, 0.732]	0.068	0.188	17.4
SA	0.894 [0.813, 0.947]	0.020	0.054	1.7
TOP	0.262 [−0.275, 0.624]	0.035	0.098	6.2
TOST	0.781 [0.613, 0.890]	0.042	0.118	14.9
**AP-GRF**				
RAW	0.209 [−0.364, 0.597]	0.070	0.194	36.8
SA	0.849 [0.732, 0.925]	0.043	0.120	3.8
TOP	0.862 [0.756, 0.931]	0.034	0.094	8.6
TOST	0.518 [0.146, 0.759]	0.045	0.125	25.7
**ML-GRF**				
RAW	0.191 [−0.449, 0.597]	0.054	0.151	40.2
SA	−0.046 [−0.906, 0.485]	0.121	0.336	9.2
TOP	0.428 [−0.013, 0.713]	0.108	0.300	48.5
TOST	0.606 [0.297, 0.803]	0.040	0.111	46.9

^1^ ICC = intraclass correlation coefficient, two-way random effects, absolute agreement, averaged five-landing score. SEM and MDC95 calculated for the averaged score. CV = mean within-athlete coefficient of variation across trials.

## Data Availability

The original contributions presented in this study are included in the article. Further inquiries can be directed to the corresponding author.

## Abbreviations

The following abbreviations are used in this manuscript:

TTS	Time to Stabilization
GRF	Ground Reaction Force
DPS	Dynamic Postural Stability
TOST	Two One-Sided Tests TTS method
ICC	Intraclass Correlation Coefficient
SEM	Standard Error of Measurement
RAW	Raw, (unprocessed) signal TTS method
SA	Sequential Average TTS method
TOP	Third-Order Polynomial TTS method
V-GRF	Vertical Ground Reaction Force
AP-GRF	Antero-Posterior Ground Reaction Force
ML-GRF	Medio-Lateral Ground Reaction Force
BW	Body Weight
IQR	Interquartile Range
LoA	Limit of Agreement
CV	Coefficient of Variation
